# P-2281. Impact of Immunosuppression on Clinical Outcomes in Critically Ill Pneumonia Patients

**DOI:** 10.1093/ofid/ofae631.2434

**Published:** 2025-01-29

**Authors:** Natalia Sanabria-Herrera, Antoni Torres, Ignacio Martin-Loeches, Luis Felipe F Reyes

**Affiliations:** Clínica Universidad de La Sabana, Chía, Colombia, Bogota, Distrito Capital de Bogota, Colombia; Hospital Clinic of Barcelona, Barcelona, Catalonia, Spain; Saint James University Hospital, Dublin, Dublin, Ireland; Universidad de La Sabana, Chía, Cundinamarca, Colombia

## Abstract

**Background:**

Nosocomial lower respiratory infections (nLRTI) are linked to poorer clinical outcomes and a significant healthcare cost. Nevertheless, there remains uncertainty regarding the risk factors for mortality among critically ill individuals, particularly those with varying degrees of immunosuppression. Thus, this study aims to comprehensively describe immunocompromised patients, assess its impact on clinical outcomes, and conduct a comparative survival analysis.

Figure 1

**Figure:**
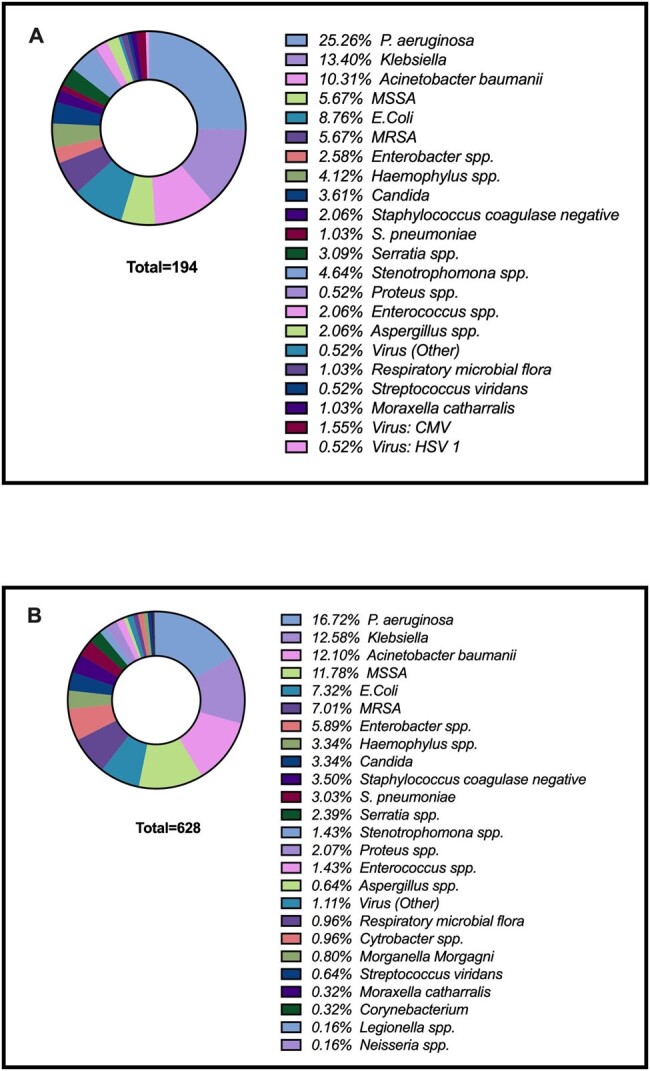

Identified microorganisms in A) the Immunosuppression group and B) Non- the Immunosuppression group

**Methods:**

This analysis used data from the prospective, multicenter, and multinational cohort developed by the European Network for ICU-related respiratory infections (ENIRRI), including 1060 adult patients diagnosed with nLRTI. Descriptive statistics were utilized to compare the immunosuppressed group against the non-immunosuppressed group, while also detailing the microorganisms predominantly identified in both groups. A survival analysis adjusted for infection severity using a Cox proportional hazards model was performed to explore mortality within these subgroups further.

Figure 2

**Figure:**
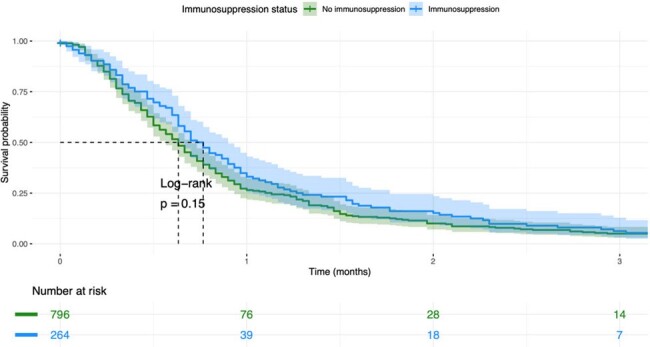

Kaplan-Meier survival curve stratified by Immunosuppression status: Probability of survival from ICU admission

**Results:**

Immunosuppression was observed in 25% of the patients (264/1060). In both cohorts, Pseudomonas aeruginosa was the predominant microorganism, particularly affecting immunosuppressed patients, (25.3% vs. 16.7%, p = 0.032), followed by Klebsiella spp and Acinetobacter baumanii (Figure 1). Interestingly, the survival analysis revealed a lower probability of survival in the non-immunosuppressed after admission to the ICU (Figure 2). Additionally, the Cox regression analysis adjusted by disease severity found that no-immunosuppressed patients had lower mortality.

Figure 3

**Figure:**
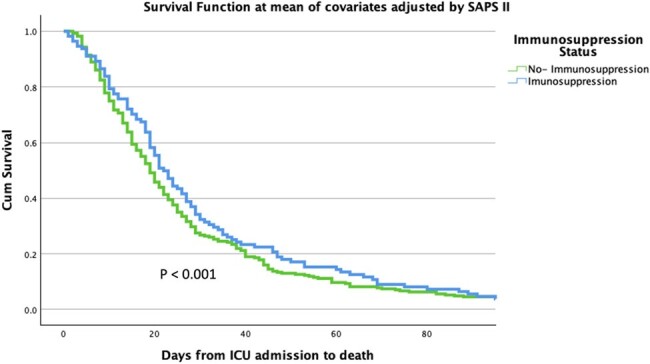

Adjusted Kaplan-Meier Survival Curve following Cox Regression: Survival Probability Adjusted for SAPS II Score.

**Conclusion:**

This description sheds light on the intricate relationship between immunosuppression, microbial etiology, and clinical outcomes in patients with nLRTI. Interestingly, immunosuppression was not associated with worse clinical outcomes, even after adjusting for disease severity.

**Disclosures:**

All Authors: No reported disclosures

